# Tympanic Membrane Rupture During Stereotaxic Surgery Disturbs the Normal Feeding Behavior in Rats

**DOI:** 10.3389/fnbeh.2020.591204

**Published:** 2020-12-01

**Authors:** María J Barahona, Joaquín Rojas, Elena A Uribe, María A García-Robles

**Affiliations:** ^1^Laboratorio de Biología Celular, Departamento de Biología Celular, Facultad de Ciencias Biológicas, Universidad de Concepción, Concepción, Chile; ^2^Centro Regional de Estudios para la Vida (CREAV), Universidad de Concepción, Concepción, Chile; ^3^Laboratorio de Enzmología, Departamento de Bioquímica y Biología Molecular, Facultad de Ciencias Biológicas, Universidad de Concepción, Concepción, Chile

**Keywords:** rats, auditory bars, food intake, recovery, body weight

## Abstract

Stereotactic surgery is a widely used procedure in neuroscience research to study the brain’s regulation of feeding behavior. In line with this notion, this study aims to assess how food consumption and feeding patterns are affected in response to the use of auditory bars that preserve or damage the tympanic membrane during stereotactic surgery. Our previous observations led us to hypothesize that the traumatic tympanic membrane rupture affects food intake and feeding patterns in rats undergoing stereotactic procedures. Thereby, female and male rats were cannulated in the third ventricle (3V) using both types of auditory bars. Post-surgical pain was assessed using the grimace scale. Food intake, meal patterns and weight gain or loss were analyzed for 5–7 consecutive days after surgery. Normal food intake, increased body weight and regular meal patterns were observed from postoperative day 2 when the stereotactic procedure was performed using auditory bars that maintain the integrity of the tympanic membrane. However, tympanic membrane rupture prevented the expected recovery of food intake and body weight. This effect was accompanied by an alteration in eating patterns, which was persistent over 7 days of recovery. Thus, tympanic membrane preservation during surgery is necessary to evaluate short-term feeding patterns. This study demonstrates auditory bars that do not damage the tympanic membrane should be used when performing stereotactic surgery for subsequent analysis of rat behavior.

## Graphical Abstract

Tympanic membrane rupture during stereotaxic surgery disturbs the normal feeding behavior in rats. **(A)** Tympanic membrane perforated. The rats were positioned in the stereotactic equipment using auditory bars that break the tympanic membrane. The rupture of the membrane prevents the body weight gain. **(B)** Tympanic membrane preserved. The rats were positioned on the stereotactic equipment using auditory bars that maintain the integrity of the tympanic membrane. The integrity of the membrane is necessary to stimulate body weight gain and increase animal welfare.

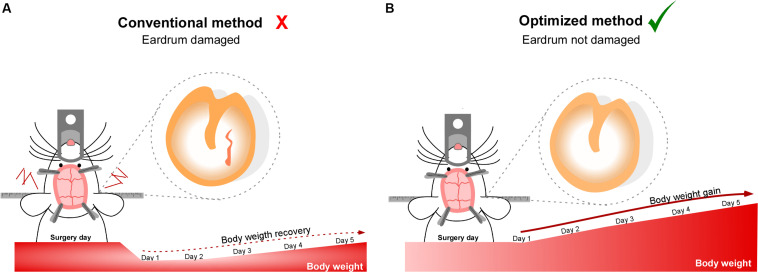

## Significance Statement

Stereotaxic procedures are widely used in behavioral studies; > 1000 behavioral studies are published annually, of which 400 used stereotaxic procedures in murine models. Nevertheless, the main of articles do not report the kind of auditory bar used to perform this surgery, especially if a feeding behavior study is described. Here, we present evidence showing that tympanic membrane rupture has a strong impact on feeding patterns in rats. Based on these findings, we strongly suggest the use of auditory bars that preserve the integrity of the tympanic membrane if a feeding behavior study is to be performed.

## Introduction

Stereotactic surgery is an experimental approach widely used in neuroscience research (Yang et al., 2018). In this technique, a needle, electrode or cannula guide is implanted with great precision into the brain, through the fixation of the subject’s head with auditory bars which allow maintain the flat head during stereotactic surgery. Therefore, correct insertion into the ear canal is a limiting step in the precision of the surgery. Currently, companies dedicated to providing supplies for stereotactic surgery offer two types of ear bars, both compatible with all rodent stereotaxic instruments. The traditional rodent ear bars have a taper at the tip to penetrate deep into the ear channel. Although they provide greater head stability, they easily damage the tympanic membrane. Tympanic membrane rupture in humans is associated with hearing loss, acute pain, increased risk of middle ear infection and spinning sensation (Orji, 2009; Gao et al., 2017), symptoms that may also appear in rodents after the eardrum traumatic perforation during stereotactic surgery. Hearing loss, acute pain and stress can strongly impact the feeding behavior of rodents (Zampini and Spence, 2012; Spence, 2015; Gonzalez-Torres and Dos Santos, 2019), where stereotactic procedures are widely used as they are an elegant tool to explore the effects of directly administered molecules in specific areas of the brain, bypassing the blood brain barrier (Jho et al., 2003).

Here, we hypothesize that traumatic tympanic membrane rupture strongly affects food intake and feeding patterns in rats undergoing stereotactic procedures. The main aim of this work is to determine which type of ear bar is most suitable for studies of eating behavior. To test our hypothesis, we evaluated food consumption, body weight and meal patterns in response to the use of auditory bars that preserve or break the tympanic membrane. We did not detect significant differences in food intake, body weight gain and eating patterns when we compared non-operated with operated rats preserving the integrity of the eardrum. However, tympanic membrane perforation induced a delay the post-surgical recovery of food intake and body weight in addition to altering eating patterns during the dark phase. Taken together, tympanic membrane preservation during stereotactic surgery is necessary to recover normal feeding pattern.

## Materials and Methods

### Ethics Statement

All studies were reviewed and approved by the Animal Ethics Committee of the Chile’s National Commission for Scientific and Technological Research (CONICYT, protocol for projects # 1180871). All animal work was approved by the appropriate Ethics and Animal Care and Use Committee of the Universidad de Concepcion, Chile. Animals were treated in compliance with the U.S. National Institutes of Health guidelines for animal care and use. Two-month-old male and female adult Sprague-Dawley rats (180–250 g) were housed in a 12-h light/dark cycle with food and water *ad libitum*. Animals were fed a standard chow diet (Lab Diet, ProLab, St. Louis, MO, United States) containing no less than 5% crude fat. Feeding behavior analysis was performed in a noise-free condition.

### Study Design

Male and female rats were age-matched and randomly assigned to experimental groups to ensure a non-biased distribution of animals. Assignment of the groups and results analysis were performed in an unblinded manner. Sample-size estimation was performed by a recursive equation, ensuring a statistical power of 0.8 and an alpha level of 0.05. Three experimental groups were estimated and used in this work: (1) experimental group 1 (*n* = 10) corresponding to non-operated rats, (2) experimental group 2 (*n* = 22) including rats operated with auditory bars that preserve the eardrum, and (3) experimental group 3 (*n* = 4–7) composed of rats operated with auditory bars that break the eardrum. Each of the experimental groups was generated through three independent experiments (*N* = 3). Since the procedure is invasive, a minimal number of animals were used for condition 3. In contrast, conditions 1 and 2 have an upper number because they are utilized as control conditions regularly in the laboratory.

### Preparation of Surgical Material

Before the surgery, surgical tools were cleaned and disinfected by sonication in a solution that contained 1% hydrogen peroxide (Difem Pharma S.A, Santiago Chile), 70% alcohol (Diprolab, Concepción Chile) and distilled water. Subsequently, the surgical pack was dried in a stove at 37°C and autoclaved in a steam autoclave. Once the surgical package was opened, the material was covered with a cloth to prevent contamination by environmental particles.

### Pre-operative Care

Rats were handled for 1 week each day to become accustomed to the researchers and experimental procedures. Three days before surgery, rats were individually housed in a cage to measure food intake and body weight. These cages record every interaction with the feeder by the interruption of an infrared sensor, which is detected by a computerized data acquisition system (VitalView, Respironics, Inc., Murraysville, PA, United States). Prior to surgery, the rats were kept at an ambient temperature of 22°C in a clean cage. All boxes were located at the same height, same light intensity and free of noise pollution. Rats were anesthetized via an intraperitoneal injection of a ketamine/xylazine (90 and 10 mg/kg, respectively) cocktail. Reflex loss was assessed through leg retraction, corneal response, and whisker movement. Subsequently, the hair was shaved from the head, and a 1% ketoprofen (10 mg/mL) and tramadol (2 mg/kg) cocktail was injected into the loin.

### Stereotactic Surgery

All surgical procedures were performed by the same researcher such that experience in performing the surgeries did not interfere with the results of this work. The surgery was performed in a room at 24°C. Two surgical protocols were compared. In protocol 1, animals belonging to experimental group 2 were positioned in the stereotactic frame (Standard Stereotaxic Instruments, RWD. Code: 68001) using auditory bars that do not damage the tympanic membrane (Ear bars, rat, RWD. Code: 68305). In protocol 2, animals belonging to experimental group 3 were positioned in the stereotactic frame (Standard Stereotaxic Instruments, RWD. Code: 68001) using auditory bars that perforate the tympanic membrane (Ear bars, rat, RWD. Code: 68301). When protocol two was carried out, the same number of animals using protocol one was operated, i.e., at the same surgery session. Both surgical procedures took between 20 and 25 min each and were executed by the same investigator. The tail was fixed to the stereotaxic frame and sheltered during the entire surgery. The skin was cleansed with chlorhexidine gluconate (Difem Pharma, 2% topical solution) with circular movements. The procedure was repeated three times. An incision was made in the skin from the middle region of the eyes to the ears using a scalpel (#10). The skin was held with forceps, and the periosteum gently removed. The skull was cleaned with 2% hydrogen peroxide, and three holes were made using a microdrill (RWD, code 78001). Subsequently, a cannula (Plastic One Inc., Roanoke, VA, 28 gauge) was implanted into the 3 V using the following stereotaxic coordinates: AP −3.14 mm, ML 0.0 mm, DV, 9.2 mm. The cannula was fixed to the skull with dental acrylic (Marche S.A Santiago, Chile) and retaining screws (Plastic One, code 00-96 × 3/32). At the end of the surgery, rats were sutured with monofilament nylon suture 5-0 (Dafilon, Braun, Melsungen, Hessen). Eyes were protected with ophthalmic ointment, and the rats were positioned over a warming mat during the whole procedure.

### Post-operative Care

Immediately after the surgery, the rats were housed individually in clean boxes at a temperature of 24°C. They were constantly monitored. Post-operative pain was evaluated for the first time at 5 h and then daily for 5 days in a row using the grimace scale (Sotocinal et al., 2011). The rats were left in the recovery room until 5 h after surgery after which they were immediately housed in the maintenance room. Animals with signs of pain after the first day of surgery received a subcutaneous injection of tramadol (2 mg/kg) and 1% ketoprofen (10 mg/mL).

### Otoscopy

Post-surgery, while the rats were still anesthetized, they underwent regular otoscopic observation on for evaluating the tympanic membrane preservation. We selected one rat by experimental condition for recording tympanic membrane preservation, exposing the ear canal under anesthesia, and the pictures were taken using a dissection stereomicroscope (Leica S4E, Wetzlar, Germany). After the photo capture, these rats were sacrificed.

### Grimace Scale

The grimace scale was evaluated in rats operated with protocols 1 and 2. Facial expression was analyzed daily for 5 days post-surgery through digital photographs. The grimace scale score was calculated as described previously (Langford et al., 2010; Sotocinal et al., 2011).

### Food Intake and Body Weight Measurements

Rats were weighed daily before the surgery and for 5 or 7 days post procedure. The percentage body weight change was calculated after 24 h. *Ad libitum* food intake was monitored by providing pre-weighted food over a defined time interval and was expressed as the amount (in grams) consumed in 24 h by 200 g of body weight. The amount of food consumed by rats that did not undergo surgery in group 1 was calculated as the amount of food ingested over 24 h normalized at 200 g body weight, and that the amount of food consumed is within the expected consumption level (Barahona et al., 2018).

### Feeding Behavior Analysis

Feeding behavior evaluated in rats was recorded by a computerized data acquisition system (VitalView, Respironics, Inc.), which individually registers the number of times each rat interacted with the feeder and the amount of time that they remained in the trough. A meal event (ME) was defined as one or more episodes longer than 5 s and no longer than 15 min, followed by a meal interval (MI) (Barahona et al., 2018). The minimum MI was defined as 10 min, as previously described (Elizondo-Vega et al., 2016; Uranga et al., 2017; Barahona et al., 2018). Microstructure parameters were evaluated. The mean meal size was determined as the total food intake (g) divided by meal frequency. The mean meal duration was calculated by dividing the total meal duration (min) by total meal events, and the eating rate was estimated by dividing total food intake (g) by total meal duration (min).

### Statistical Analyses

Results were expressed as mean standard error (SEM). Subjects were neither excluded from the experiment nor the statistical analysis. D’agostino-pearson omnibus normality test was performed to evaluate the normal data distribution. For statistical analysis of food intake, each recovery day was compared to its respective control (Experimental group 1: non-operated rats). For the statistical analysis of body weight, recovery day 1 was compared to days 2, 3, 4, and 5 post-surgery. Non-parametric data was analyzed using a Kruskal-Wallis test followed by Dunn’s *post-hoc* multiple comparisons test. Two-way ANOVA followed by Bonferroni *post-hoc* multiple comparisons tests were performed for parametric data analysis. All the results of the statistical tests are found in Table 1. The statistical analyses were performed using GraphPad Prism 5.0 Software (GraphPad Software Inc., San Diego, CA, United States).

**TABLE 1:** Statistical results for unpaired *t*, Kruskal-Wallis, one-way and two-way ANOVA test analyses.

Figure number	Test used	n	Data reported	P-value	F(DFn, DFd)	t	K	DF
Figure 1D	Two way-ANOVA	n: 3–4	Mean ± SEM	Interaction: P = 0.2716	Interaction F(5, 30) = 1.340			Interaction: 5
				Column factor: P = 0.0514	Column factor: F(1, 30) = 4.116			Column factor: 1
Figure 2A	Two way-ANOVA	n: 7–26	Mean ± SEM	Interaction: P = 0.0001	Interaction F(8, 188) = 5.983			Interaction: 8
				Column factor: P = 0.0001	Column factor: F(2, 188) = 91.68			Column factor: 2
Figure 2B	Two way-ANOVA	n: 7–26	Mean ± SEM	Interaction: P = 0.0001	Interaction F(4, 144) = 9.825			Interaction: 4
				Column factor: P = 0.0182	Column factor: F(1, 144) = 5.711			Column factor: 1
Figure 2C	Unpaired t test	n: 23	Mean ± SEM	P = 0.0001		t = 4.620		
Figure 2D	Unpaired t-test	n:7	Mean ± SEM	P = 0.7791		t = 22.00		
Figure 3A	Kruskal-Wallis test	n:4–7	Mean ± SEM	P = 0.0002			K = 33.47	
Figure 3B	Kruskal-Wallis test	n:4–7	Mean ± SEM	P = 0.0001			k = 41.40	
Figure 3C	Two way-ANOVA	n:4–7	Mean ± SEM	Interaction: P = 0.0001	Interaction F(14, 112) = 4.220			Interaction: 14
				Column factor: P = 0.0001	Column factor: F(2, 112) = 16.18			Column factor: 2
Figure 3D	Two way-ANOVA	n:4–7	Mean ± SEM	Interaction: P = 0.0001	Interaction F(14, 104) = 4.363			Interaction: 14
				Column factor: P = 0.0001	Column factor: F(2, 104) = 16.48			Column factor: 2
Figure 3E	Two way-ANOVA	n:4–7	Mean ± SEM	Interaction: P = 0.0001	Interaction F(14, 104) = 3.864			Interaction: 14
				Column factor: P = 0.0014	Column factor: F(2, 104) = 7.030			Column factor: 2
Figure 3F	Two way-ANOVA	n:4–7	Mean ± SEM	Interaction: P = 0.0009	Interaction F(14, 96) = 2.942			Interaction: 14
				Column factor: P = 0.2162	Column factor: F(2, 96) = 1.556			Column factor: 2
Figure 3G	Two way-ANOVA	n:4–7	Mean ± SEM	Interaction: P = 0.0839	Interaction F(14, 93) = 1.636			Interaction: 14
				Column factor: P = 0.2253	Column factor: F(2, 93) = 1.514			Column factor: 2
Figure 4A	Kruskal-Wallis test	n: 4–6	Mean ± SEM	P = 0.5599			k = 1.559	
Figure 4B	Kruskal-Wallis test	n:4–6	Mean ± SEM	P = 0.5924			K = 1.140	
Figure 4C	Kruskal-Wallis test	n: 4–6	Mean ± SEM	P = 0.0449			k = 5.843	
Figure 4D	Kruskal-Wallis test	n:4–6	Mean ± SEM	P = 0.0101			K = 7.896	
Figure 4E	Kruskal-Wallis test	n: 4–6	Mean ± SEM	P = 0.0054			k = 8.643	
Figure 5a	Unpaired test	n:4–6	Mean ± SEM	P = 0.0095		t = 0.0		
Figure 5B	Unpaired test	n: 4–6	Mean ± SEM	P = 0.1143		t = 6.000		
Figure 5C	Two way-ANOVA	n: 4–6	Mean ± SEM	Interaction: P = 0.0625	Interaction F(4, 40) = 2.439			Interaction: 4
				Column factor: P = 0.0173	Column factor: F(1, 40) = 6.169			Column factor: 1
Figure 5D	Unpaired test	n: 4–6	Mean ± SEM	P = 0.8252		t = 11.00		
Figure 5E	Unpaired test	n: 4–6	Mean ± SEM	P = 0.3476		t = 10.00		
Figure 5F	Unpaired test	n: 4–6	Mean ± SEM	P = 0.3448		t = 9.00		

**n, number of animals used in this study; SEM, standard error of the mean; F, F statistic; DFn, numerator degrees of freedom; DFd, denominator degrees of freedom; K, Kruskal-Wallis statistic; DF, degrees of freedom, t, unpaired t-test statistic. D’Agostino and Pearson omnibus normality test was applied to evaluate the samples normality. Two groups comparison was performed using a unpaired t-test. The comparison between more of one non-parametric groups was performed using a Kruskal-Wallis test, while comparisons between parametric samples were made through One way-ANOVA. Comparisons between two groups and two variables were made by applying a Two way-ANOVA. No animals were excluded from the statistical analysis.**

## Results

### The Pain in Rats Was Mainly Caused by the Surgical Procedure and Not by Tympanic Membrane Rupture

**FIGURE 1:**
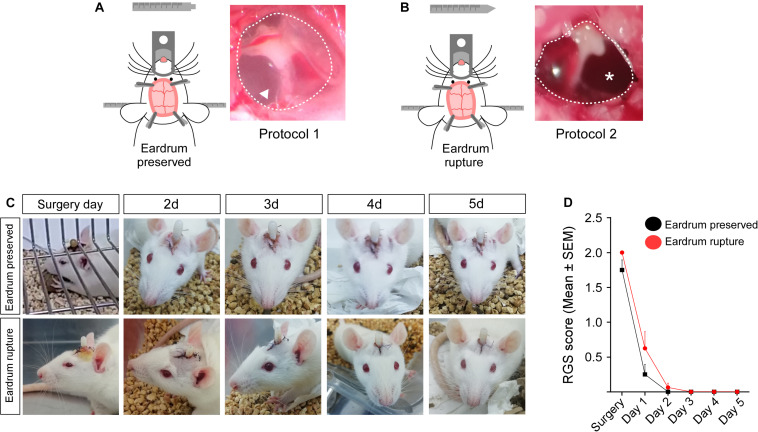
Grimace scale score in rats operated with bars that preserve or damage the tympanic membrane. Tympanic membrane of rats operated using protocol 1 **(A)** or protocol 2 **(B)**. **(C)** Facial photographs of rats operated with auditory bars that preserve or damage the tympanic membrane. Facial photographs were realized during 5 days of post-surgery recovery. **(D)** Grimace scale score obtained for 5 days post-surgery in rats operated with auditory bars that preserve (black lines; n: 3) or break the tympanic membrane (red bars; n: 4). Data are expressed as mean ± SEM. Two-way ANOVA (Bonferroni’s multiple comparisons test).

At the end of the stereotaxic procedures, we evaluated the integrity of the tympanic membrane in rats operated with blunt-tip (protocol 1) or cone-tip (protocol 2). As showed in Figure 1A, blunt-tipped auditive bars do not break the tympanic membrane (arrow). However, cone-tip auditive bars, used mostly for stereotaxic procedures, cause the rupture in the tympanic membrane (Figure 1B, asterisk). Because tympanic membrane rupture can cause acute pain during the first hours after trauma (Orji, 2009), with consequences in the feeding behavior, we evaluated the level of pain, through the application of the grimace scale, in rats operated with protocols 1 and 2. The grimace scale indicates that rats operated under both protocols did not show significant differences during the 5 days of post-surgical recovery. Both groups exhibited the highest RGS score at 5 h post-surgery (Figures 1C,D). Therefore, the results suggest that pain in rats was mainly caused by surgery and was not associated with tympanic membrane rupture.

### Preservation of the Tympanic Membrane Favors the Recovery of Food Intake in Rats Undergoing Stereotaxic Surgery

**FIGURE 2:**
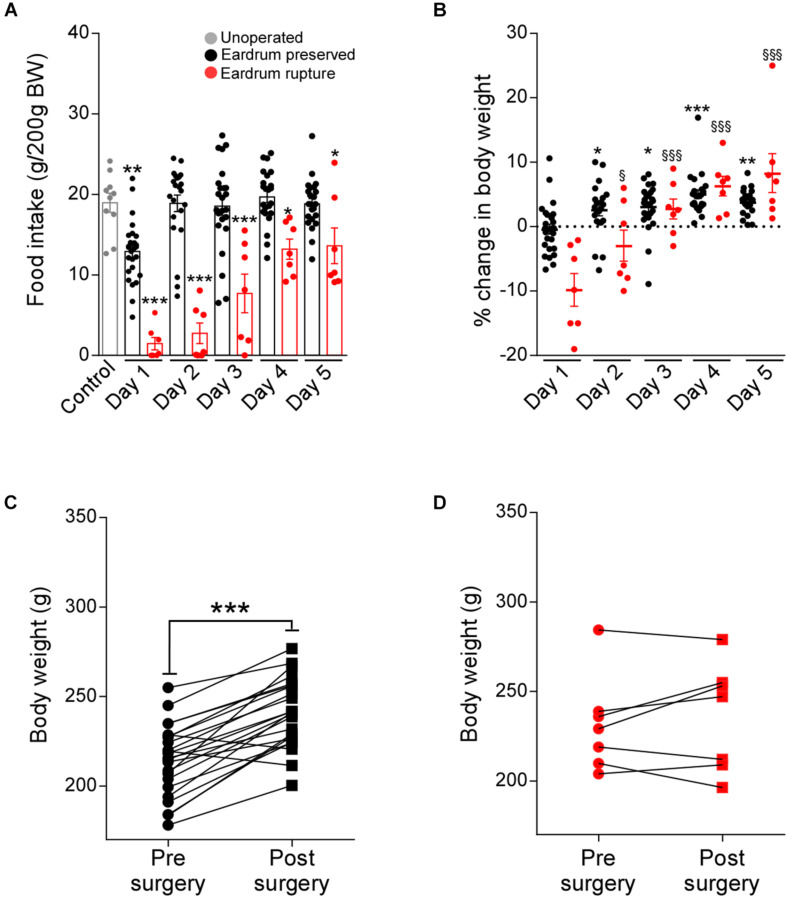
Traumatic tympanic membrane perforation prevents normal recovery of food intake and body weight after surgery. Total food intake (g/200 g BW) **(A)** and percentage (%) of change in body weight (BW) **(B)** at 24 h after feeding. Food intake and body weight were measured daily during 5 days of recovery in rats cannulated using protocol 1 (n: 22, black bars) or using protocol 2 (n: 7, red bars). Non-operated rats were used as control (n: 10, gray bars). The body weight of non-operated rats was represented as a dotted line. Body weight (g) at pre and post-surgical state in rats cannulated using protocol 1 **(C)** or using protocol 2 **(D)**. Pre-surgical body weight (g) was measured 1 day before surgery, post-surgical body weight (g) was measured at day 5 post-surgery. Data are expressed as mean ± SEM. Food intake and % of the change in body weight were analyzed through two-way ANOVA (Bonferroni’s multiple comparisons test). For statistical analysis of food intake, each recovery day was compared to non-operated rats (asterisks). For the statistical analysis of change of body weight, recovery day 1 was compared to days 2, 3, and 5 post-surgery of protocol 1 (asterisks) or protocol 2 (§). A comparison between the two groups was analyzed through unpaired *t*-test (asterisks). The experiment is the result of three independent repetitions (N: 3). *p < 0.05, **p < 0.01, ***p < 0.001. ^§^ *p* < 0.05, ^§§^ *p* < 0.01, ^§§§^ *p* < 0.001.

Previous studies have shown that feeding is a multifactorial physiological mechanism that depends on the brain integration of endocrine signaling (e.g., leptin and ghrelin); nutrient sensing (e.g., glucose, lipids, and amino acids) and sensory signals, such as taste, vision and hearing (Spence, 2015). In line with this hypothesis, we evaluate whether the ruptured eardrum, affects feeding behavior. Total food intake and percentage change in body weight were analyzed during 5 days post-surgery in rats submitted to protocol 1 (Figures 2A–C; black dots) and protocol 2 (Figures 2A,B,D; red dots). Control rats (non-operated; gray dots) eat an average of 18 g for 24 h. Rats operated using protocol 1 showed a significantly lower food intake only on day 1 post-surgery than non-operated rats (Figure 2A). Body weight was rapidly recovered from day 2 post-surgery (Figure 2B). Indeed, body weight increased significantly at 5 days post-surgery (Figure 2C). However, using protocol 2, different feeding behavior was observed. Food intake was lower than non-operated rats from day 1 to day 5 post-surgery (Figure 2A). However, the% of the change in body weight was significantly increased from day 2 post-surgery (Figure 2B); despite this, on the 5 days after surgery, they only recovered the lost weight without additional gain weight (Figure 2D). Therefore, preservation of the tympanic membrane favors the recovery of food intake and the gain of body weight in rats undergoing stereotaxic surgery.

### Rupture of the Tympanic Membrane Affects the Establishment of Satiety

**FIGURE 3:**
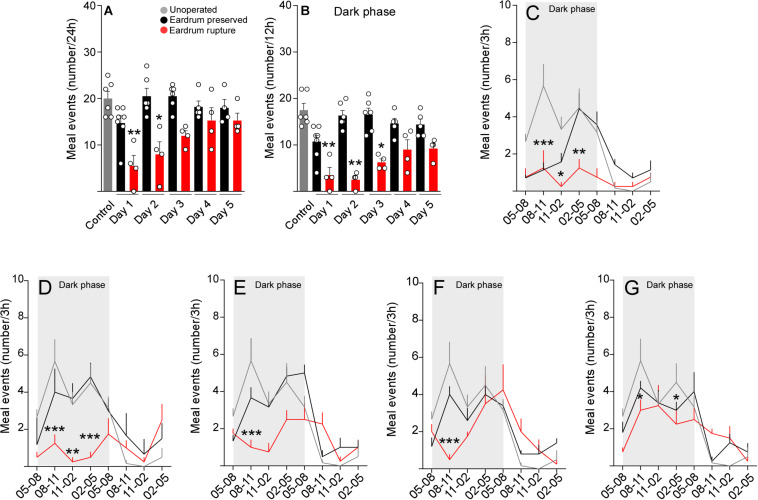
Tympanic membrane integrity is required to maintain normal feeding patterns. Total meal events (number) at 24 h **(A)** and 12 h **(B)** after 1, 2, 3, 4, and 5 days post-surgery in cannulated rats following the protocol 1 (black bars) or protocol 2 (red bars). **(C–G)** Pattern of eating events in 24 h after 1 day **(C)**, 2 days **(D)**, 3 days **(E)**, 4 days **(F)**, and 5 days **(G)** of recovery in cannulated rats using the protocol 1 (black line) or protocol 2 (red line). Meal events were analyzed as total cumulative over 3 h during the 24-h feeding period. As a control, non-cannulated rats were used (gray bars). The data are expressed as mean ± SEM. Total meal events were analyzed through Kruskal-Wallis test (Dunn’s multiple comparisons test), *p < 0.05, **p < 0.01. Pattern of eating events were analyzed through a two-way ANOVA (Bonferroni’s multiple comparisons test), *p < 0.05, **p < 0.01, ***p < 0.001.

Satiety is a mechanism causing a delay in the initiation of a new meal i.e., prolongs the time interval until the next meal is voluntarily initiated (Elizondo-Vega et al., 2016; Uranga et al., 2017; Barahona et al., 2018; Elizondo-Vega et al., 2019). In line with this concept, we evaluated the number of meal events in cannulated rats with protocols 1 or 2 during 5 post-operative days. As seen in Figure 3A, rats cannulated with protocol 2 showed a significant minor number of feeding events during days 1 and 2 after surgery in comparison with the control group. It is relevant to mention that a significant reduction in the number of events was seen only in the dark phase of feeding (12 h) (Figure 3B), a stage in which rats carry out the process of feeding. For a more detailed analysis, we evaluated the kinetics of feeding events. On the first day after surgery, using the auditory bars that damage the eardrum (protocol 2), we detected a significantly lower food intake in all dark phase (Figure 3C). While using the auditory bars that preserve the eardrum (protocol 1), the reduction of food intake was significantly lower only between 5 and 11 PM. From post-surgical day 2, rats cannulated with protocol 1 recovered their typical feeding pattern without showing significant differences with the rats that did not undergo surgery (Figures 3D–G, black line). Nevertheless, rats cannulated with protocol two during the five days of recovery had an abnormal feeding pattern, which included a minor food intake at the start of the dark phase (Figures 3D–G, red line). Altogether, these results suggest that the integrity of the tympanic membrane is essential to restore the feeding patterns after stereotaxic surgery.

**FIGURE 4:**
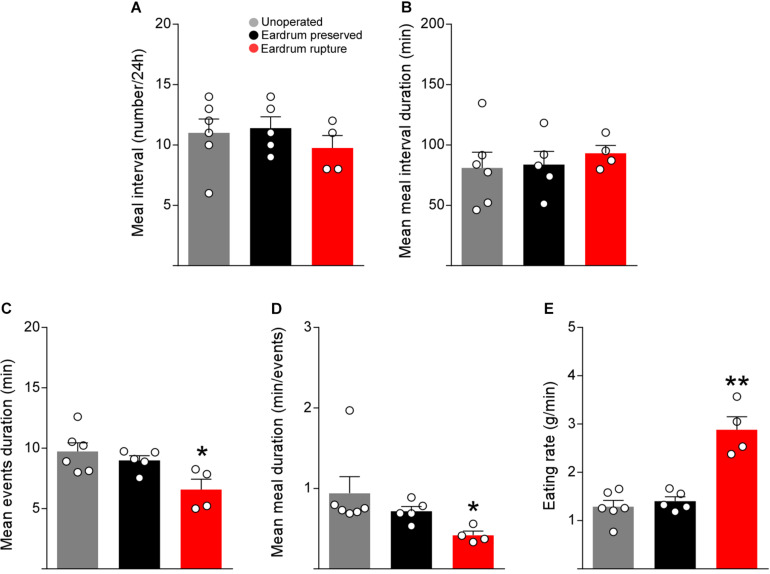
Tympanic membrane rupture alter the feeding microstructure parameters. Total meal interval (number) **(A)**, mean meal interval duration (min) **(B)**, meal events duration (min) **(C),** mean meal duration (min/events) **(D)**, eating rate (g/min) **(E)** at day 5 post-surgery in cannulated rats following the protocol 1 (black bars) or protocol 2 (red bars). As a control, non-cannulated rats were used (gray bars). The data are expressed as mean ± SEM. Kruskal-Wallis test (Dunn’s multiple comparisons test), *p < 0.05, **p < 0.01.

To determine if the microstructure indicators of feeding normalize after surgery, we evaluated the number and duration of the intervals between meals, duration of events, the period of the meals, and feeding rate in manipulated rats with protocols 1 and 2 at 5 days post-surgery. The number and duration of intervals between meals were not altered in rats manipulated with both protocols (Figures 4A,B). However, rats manipulated with protocol 2 presented a significant decrease in the duration of meals (Figures 4C,D) and a significant increase in the rate of feeding (Figure 4E), suggesting that 5 days of recovery after surgery are not enough to normalize the feeding behavior when using bars that break the eardrum.

### The Rupture of the Tympanic Membrane Affects the Feeding Behavior

**FIGURE 5:**
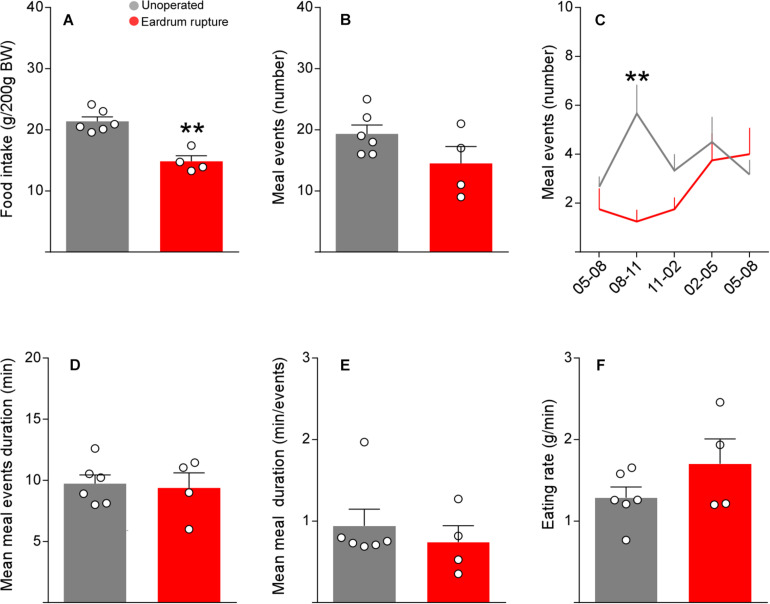
Impaired feeding behavior at day-7 post-surgery after tympanic membrane rupture. Total food intake (g/200 g BW) **(A)**, meal events (number) **(B)**, meal events in the 12 h of the dark phase of feeding **(C)**, mean meal events duration (min) **(D)**, mean meal duration (min/events) **(E)** and eating rate (g/min) **(F)** at day 7 post-surgery in control rats (n: 6) (gray bars) or in rats operated using the protocol 2 (red bars) (n: 4). The data is presented as the mean ± SEM. Two-way ANOVA (Bonferroni’s multiple comparisons test) and unpaired *t-*test. **p < 0.01.

Previously, we showed that 5 days of recovery after surgery are not enough to restore the normal control of feeding behavior when rats undergo surgery using auditory bars that break the eardrum. To determine if 7 days of recovery are enough to normalize feeding, food intake, feeding events, kinetics of feeding events, duration of feeding events, meal duration and feeding rate in control rats and rats cannulated with protocol 2 were analyzed. Interestingly, rats manipulated with protocol 2 showed a significant decrease in the amount of food consumed on day 7 of recovery (Figure 5A). Moreover, although the number of total events over 24 h did not differ with that of the control group (Figure 5B), rats manipulated with protocol 2 presented a significant loss in the first peak of food intake between 20.00 and 23.00 h (Figure 5C). These data suggest that even with 7 days of recovery, rats do not recover their normal feeding kinetics, which may strongly affect any study of feeding behavior using this protocol. However, microstructure indicators of feeding are restored after 7 days of post-surgical recovery (Figures 5D–F). Altogether, the results show that rupture of the tympanic membrane affects short- and long-term feeding behavior. Therefore, the use of blunt-tip auditive bars in stereotactic procedures are suggested if a feeding behavior analysis is required.

## Discussion

A large number of sensory science studies have been published, demonstrating that auditive signals play an important role in the multisensory perception of food attributes, such as crunchy, carbonated and even creamy (see Spence, 2015). Even though some of these perceptions may initially seem oral-somatosensory in nature, our experience in mouth may be radically changed by modifying chewing sounds, which could be essential in rodents.

Alterations in feeding underly various illnesses, such as obesity and nervous anorexia, which are characterized by changes in food intake and may be induced by specific diets (e.g., obesity induced by a high-fat diet). In rodent feeding disorder models, researchers often link food intake to the increase in body weight or other changes in physiology. Nevertheless, the explanatory power of these relations depends on the ability of quantifying food intake with precision. Even if measuring food intake is a simple concept, achieving precise measurements in research studies remains a technical challenge.

In this study, we provide evidence showing that selection of auditory bars can impact the recovery time of intake and maintenance of integral feeding patterns in cannulated rats. Specifically, we have demonstrated that the use of auditory bars that break the tympanic membrane prevents the normal recovery of food intake, resulting in loss of approximately 4 and 10% body weight during the first 2 post-operative days, which does not change by increasing the recovery time. Similar results were reported by Fornari et al. (2012). The authors presented a refined protocol of stereotactic surgery which included changes in anesthesia, but kept the use of ear bars that damage the eardrum. Even though refinement in the surgical protocol produced a decrease in body weight loss in comparison with a standard protocol, body weight was also reduced during the first 3 days of recovery after surgery. Furthermore, these authors state that approximately 3% of the animals did not survive the surgery. Here, we propose for the first time that the use of auditive bars that do not break the eardrum prevent appetite loss, increase body weight gain, and help maintain feeding patterns without affecting the survival of animals. Also, rats manipulated with this protocol eat right after they wake up from the surgical procedure, and their appetite remains constant during the recovery days, which resulted in an increase of body weight from day 2 after surgery, demonstrating the effectiveness of the protocol. It is important to mention that our study has limitations since it was carried out in rats. However, we believe that the data obtained are of great relevance for neuroscience research, and we strongly suggest its use in different species, such as rats and guinea pigs, even when the goal of the researchers is different from the one proposed in this study.

Rupture of the tympanic membrane affects the recovery of normal feeding behavior. Even if the grimace scale data suggests that the pain caused by the rupture does not seem to be involved, we cannot discard that the sensitivity of this method may not be high enough to detect small differences. It has been proposed that eating is a multisensory behavior (Spence, 2015; Scarpina et al., 2016) and that hearing has a role in the perception of flavor (Zampini and Spence, 2012). Hearing can be recovered after the regeneration of the tympanic membrane, which happens 7–10 days after the injury in rats, and the process of healing is completed after day 14 (Araujo et al., 2014). Also, other factors, such as stress and/or vertiginous syndromes, caused by the rupture of the tympanic membrane can delay feeding recovery. We strongly believe this data could be useful to improve the protocols that evaluate the nutritional behavior since the intake parameters were comparable with animals under surgery. In addition to this, it is possible our protocol could help obtain reliable results for neuroscience studies using stereotactic procedures. This is not only beneficial to animals, but it also has the potential to increase the quality of results at the same time that it meets the international standards of bioethics.

## Data Availability Statement

The raw data supporting the conclusions of this article will be made available by the authors, without undue reservation, to any qualified researcher.

## Ethics Statement

All studies were reviewed and approved by the Animal Ethics Committee of the Chile’s National Commission for Scientific and Technological Research (CONICYT, protocol for projects # 1180871). All animal work was approved by the appropriate Ethics and Animal Care and Use Committee of the Universidad de Concepcion, Chile.

## Author Contributions

MB and MG-R conceived the experiments, analyzed the data, and wrote the manuscript. MB, JR, and MG-R designed the experiments. MB and JR performed the experiments. MG-R, EU, and JR contributed reagents, materials, and analysis tools. MB, MG-R, JR, and EU critically revised the manuscript. All authors have approved the final version of the manuscript and agreed to be accountable for all aspects of the work in ensuring that questions related to the accuracy or integrity of any part of the work are appropriately investigated and resolved.

## Conflict of Interest

The authors declare that the research was conducted in the absence of any commercial or financial relationships that could be construed as a potential conflict of interest.
